# Detection of virulence and multidrug resistance operons in *Pseudomonas aeruginosa* isolated from Egyptian Baladi sheep and goat

**DOI:** 10.14202/vetworld.2019.1524-1528

**Published:** 2019-10-04

**Authors:** A. N. Dapgh, A. S. Hakim, H. A. Abouelhag, A. M. Abdou, E. A. Elgabry

**Affiliations:** 1 Department of Bacteriology, Animal Health Research Institute, Dokki, Giza, Egypt; 2 Department of Microbiology and Immunology, National Research Centre, 33 Bohouth Street, 12622 Dokki, Cairo, Egypt

**Keywords:** drug resistance, goat, *Pseudomonas aeruginosa*, sheep, virulence

## Abstract

**Background:**

Pseudomonas aeruginosa is a pit of an enormous group of free-living bacteria that are able to live everywhere and suggested to be the causative agent of great scope of acute and chronic animal infections.

**Aim:**

The current study was carried out to illustrate the prevalence of *P. aeruginosa* in small ruminants and existence of some virulence operons as well as its antimicrobial resistance.

**Materials and Methods:**

A total of 155 samples from sheep and 105 samples from goats (mouth abscesses, fecal swabs, nasal, tracheal swabs, and lung tissue) were collected for bacteriological study, existence of some virulence expression operons with the study of their sensitivity to the antimicrobials using disc diffusion and presence of *mexR* operon which is responsible for multidrug resistance (MDR).

**Results:**

The bacteriological examination revealed that *P. aeruginosa* was isolated from nine out of 155 samples from sheep (5.8%) and four isolates out of 105 samples from goat (3.8%). It is found that 12 (92.3%), 10 (76.9 %), and 8 (61.5%) of *P. aeruginosa* isolates harbored hemolysin phospholipase gene (*pcl* H), gene (*exo* S), and enterotoxin gene (*tox* A), respectively. The results of antibiotic sensitivity test showed that all tested isolates were resistant to ampicillin, bacitracin, erythromycin, streptomycin, tetracycline, trimethoprim-sulfamethoxazole, and tobramycin but sensitive to ciprofloxacin and norfloxacin. The MDR (*mex* R) operon was existed in all isolates.

**Conclusion:**

There is a growing risk for isolation of virulent MDR *P. aeruginosa* from sheep and goat illness cases, and this should be regarded in the efficient control programs.

## Introduction

*Pseudomonas aeruginosa* is a Gram-negative, encapsulated, nonsporulated, and strict aerobic motile rod. It is an opportunistic pathogen, widely exists in various ecosystems and believed to be implemented in several serious human and animal diseases [1,2]. *P. aeruginosa* causes numerous diseases in sheep and goats; respiratory illness, which is one of the major issues particularly pneumonia, associated with physical and physiological stress, leading to significant mortality rates, and increased economic loss [3]. A number of mastitis cases and the pathogen can reside in the udder for many years [4]. Moreover, *P. aeruginosa* infection may lead to urogenital disorders, gastrointestinal illness sinusitis, and osteomyelitis [5-8].

The organism declares plenty of virulence agents, which share in its pathogenicity. These comprise enterotoxins, exocytotoxins, and toxins produced by protein secretion systems, as a result of expression of certain virulence operons. Consequently, many of these have been implemented in infection, septicemia, and fatal condition [9,10]. The mortality rate is usually higher than bacteremia sourced with other Gram-negative pathogens due to its ability to secrete these several products that after successive colonization can induce extensive tissue damage, bloodstream invasion, and dissemination [11]. Through all resistant pathogens, the condition is most significant for *P. aeruginosa* as its incidence of resistance to antimicrobial agents in continuous increasing and has been accounted worldwide [12]. This critical ability of the pathogen could be attributed to the existence of the unusually restricted outer membrane permeability which acts as a safeguard barrier for antibiotics to overcome. Besides that, there were other secondary intrinsic factors as energy-dependent multidrug efflux and chromosomally encoded periplasmic beta-lactamase [13].

This study was conducted to address its isolation and identification from ovine and caprine population, with stressing on its toxigenic expressed operons as well as the associated multidrug resistance (MDR) property.

## Materials and Methods

### Ethical approval

As per CPCSEA guidelines, a study involving clinical samples does not require the approval of the Institute Animal Ethics Committee.

### Samples

A total of 260 different samples were collected from sheep (155) and from goat (105) selectively suffering from respiratory manifestation (40 from sheep, and 22 from goats) in triplicate as nasal, tracheal swabs, and lung tissues. Also, fecal samples were collected from diarrheic (14 sheep and 23 goats) and gathered swabs from skin lesion with abscesses (21 sheep and 16 goats). The samples were gathered from scattered local farms or owners in Great Cairo and Delta rural areas. The samples were gathered from September 2017 to April 2018. The samples were bacteriologically examined and the selected colonies were biochemically identified [14].

### Antimicrobial sensitivity assay

The susceptibility of the isolates to various antibiotics was achieved using the diffusion technique [15], the following antibiotic discs were used; amikacin (30 mg), ampicillin (10 µg), bacitracin (10 µg), chloramphenicol (30 µg), erythromycin (15 µg), gentamycin (10 µg), streptomycin (10 µg**)**, tetracycline (30 µg), trimethoprim-sulfamethoxazole (2.25/7.75 µg), tobramycin (30 µg), ciprofloxacin (5 µg), and norfloxacin (10 µg) [16].

### DNA extraction

DNA extraction from the samples was performed using the QIAamp DNA Mini kit (Qiagen, Germany, GmbH) with modifications from the manufacturer’s recommendations. Briefly, 200 µl of the sample suspension was incubated with 10 µl of proteinase K and 200 µl of lysis buffer at 56°C for 10 min. After incubation, 200 µl of 100% ethanol was added to the lysate. The sample was then washed and centrifuged following the manufacturer’s recommendations. Nucleic acid was eluted with 100 µl of elution buffer provided in the kit.

### Polymerase chain reaction (PCR) amplification using oligonucleotide primers

Primers used were supplied from Metabion (Germany), as shown in Tables-1 and 2 [16,17]. Primers were utilized in a 25 µl reaction containing 12.5 µl of Emerald Amp Max PCR Master Mix (Takara, Japan), 1 µl of each primer of 20 pmol concentrations, 4.5 µl of water, and 6 µl of DNA template. The reactions were performed either uniplex (*mex* R) or multiplex (*exo* S, *pcl* H, and *tox* A), as described in Tables-1 and 2 [17,18], in a T3 Biometra thermal cycler.

**Table 1:** Uniplex PCR: Primers sequences, target operons, amplicon sizes, and cycling conditions.

Target operon	Primers sequences	Amplified segment (bp)	Primary denaturation	Amplification (35 cycles)			Final extension	Reference

				Secondary denaturation	Annealing	Extension		
*mex* R	GCGCCATGGC CCATAT TCAG	637	94°C 5 min	94°C 45 s	57°C 45 s	72°C 45 s	72°C 10 min	[16]
	GGCATTC GCC AGTAAGCGG							

PCR=Polymerase chain reaction

**Table 2:** Multiplex PCR: Primers sequences, target genes, amplicon sizes, and cycling conditions.

Target gene	Primers sequences	Amplified segment (bp)	Primary denaturation	Amplification (30 cycles)			Final extension	Reference

				Secondary denaturation	Annealing	Extension		
*exo* S	CCTTCCCT CCTTCCCCC CGGCGATCTGGA AAAG AAATG	270	95°C 5 min	95°C 30 s	58°C 30 s	72°C 50 s	72°C 10 min	[17]
	CATCCTCA GGCG TACATCCT							
*pcl* H	GAAGCCAT GGGCT ACTTCAA	307						
	AGAGTGA CGAGG AGCGGTAG							
*tox* A	ATGGTGTA GATC GGCGACAT	433						
	AAGCCTTC GACC TCTGGAAC							

PCR=Polymerase chain reaction

### Analysis of the PCR products

The products of PCR were electrophoresed on 1.5% agarose gel (Applichem, Germany, GmbH) in 1× TBE buffer at room temperature using gradients of 5V/cm. For gel analysis, 15 µl of the products were loaded in each gel slot. A 100 bp DNA Ladder (Qiagen, Germany, GmbH) was used to determine the fragment sizes. The gel was photographed by a gel documentation system (Alpha Innotech, Biometra) and the data were analyzed through computer software. *P. aeruginosa* (ATCC 27853) was used as positive controls and distilled water (Merck, Germany) was used as a negative control in all PCR reactions.

*P. aeruginosa* (ATCC 15442) was used as a positive control while *Staphylococcus aureus* (ATCC 25923) was used as a negative one.

## Results and Discussion

*P. aeruginosa* is an environmental ubiquitous nonpathogenic saprophyte present in animate and inanimate media. Otherwise, in many statuses, this microorganism becomes a primary pathogen and is encountered in severe localized or generalized infections in human, animals, and birds particularly among immune-compromised hosts [19]. The organism was implicated in multiple diseases in sheep and goat; respiratory manifestation, diarrhea, skin abscess, and the severity of the condition may result in death cases [20,21].

In this study, as shown in Table-3, it was found that *P. aeruginosa* was successfully isolated from (6/40) sheep and (2/22) goat with respiratory manifestation with an incidence of 15% and 9.1%, respectively. The isolates were recovered from 3 (7.5%) sheep and 2 (9.1%) goat nasal swabs. Furthermore, the organism was recovered from 2 (5%) tracheal swabs and 1 (2.5%) lung tissue belonged only to sheep samples.

**Table 3:** Prevalence of *Pseudomonas aeruginosa* recovered from living and slaughtered sheep and goat.

Groups	Type of samples	Sheep			Goat		
	
		No. of samples	Positive samples number	Positive samples %	No. of samples	Positive samples number	Positive samples %
I–animals with respiratory manifestation	Nasal swabs	40	3	(7.5)	22	2	(9.1)
	Tracheal swabs	40	2	(5)	22	0	0
	Lung tissues	40	1	(2.5)	22	0	0
II–animals with skin lesions	Wound and abscesses	21	2	(9.5)	16	1	(6.25)
III–diarrheic animals	Fecal	14	1	(7.1)	23	1	(4.3)
Total		155	9	(5.8)	105	4	(3.8)

These average percentage results agreed with that formerly reported [22,23] while the lower incidence was 3.6% [24] and one necropsied goat [25]. In contrast, higher isolations were determined [26]. Two *P. aeruginosa* isolates were recovered from diarrheic sheep 1/14 (7.1%) and goat 1/23 (4.3%), respectively. On the other hand, the animals infected with wound and abscesses, the percentage of isolated *P. aeruginosa* from sheep was 2/21 (9.5%) and goat 1/16 (6.25%), respectively. These obtained data close to that stated by Hears *et al*. [27], who isolated three strains *P. aeruginosa* from one of the infected sheep flocks. Abd El-Rahman [28] deduced that the incidence of *P. aeruginosa* recovered from diarrheic sheep was higher than that obtained among specimens of other infected animals. Furthermore, current results less than that obtained by Alkeshan [29]**,** who isolated *P. aeruginosa* from abscesses with an incidence (6.18%).

The unselective use of antibiotics is potentially leading to a higher incidence of infections with resistant microorganisms such as *P. aeruginosa*, worse which may be transmitted from animal to human complicating the treatment of human diseases [30]. Regarding, the antimicrobial sensitivity agar test shown in (Table-4); it was noticed that the isolates were lack of susceptibility to many tested antimicrobial agents. The primary mechanism of the microorganism’s resistance relies on its ability to shut out various agents rather than the production of antibiotic inactivating enzymes. Consequently, most of antibiotics are of limited value in the treatment of *P. aeruginosa* infection in animals [31].

**Table 4:** Antibiotic sensitivity test of (13) *Pseudomonas aeruginosa* strains isolated from sheep and goat.

Antimicrobial agents	Sensitive	Resistant
	
	*n* (%)	*n* (%)
Amikacin	3 (23.1)	10 (76.9)
Ampicillin	0 (0)	13 (100)
Bacitracin	0 (0)	13 (100)
Chloramphenicol	2 (15.4)	11 (84.6)
Erythromycin	0 (0)	13 (100)
Gentamicin	4 (30.8)	9 (69.2)
Streptomycin	0 (0)	13 (100)
Tetracycline	0 (0)	13 (100)
Trimethoprirn/sulfamethoxazole	0 (0)	13 (100)
Tobramycin	0 (0)	13 (100)
Ciprofloxacin	13 (100)	0 (0)
Norfloxacin	12 (92.3)	1 (4.5)

The results indicated that the isolates were susceptible to both ciprofloxacin 13 (100%) and norfloxacin 12 (92.3%), while completely resistant to ampicillin, bacitracin, erythromycin, streptomycin, tetracycline, trimethoprim-sulfamethoxazole, and tobramycin, most of the isolates were resistant to amikacin, chloramphenicol, and gentamycin. These findings agreed with the other previous studies which illustrated that the organism is resistant to all used antibiotics except quinolones [32-35].

**Figure-1:**
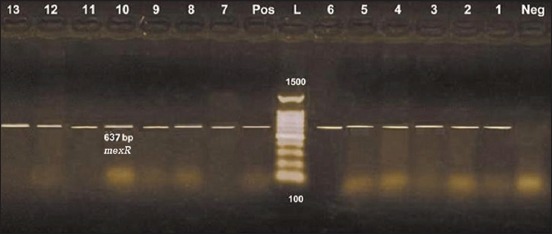
Uniplex polymerase chain reaction detection of antibiotic-resistant *mexR* operon in *Pseudomonas aeruginosa* isolates showing: L: 100 bp DNA ladder. Lanes 1-13: *P. aeruginosa* isolates. Lane Pos.: Positive control; amplification of 637 bp represented *mexR*. Lane Neg.: Negative control.

Assorted chromosomally encoded efflux systems and outer membrane porins have been distinguished as essential contributors to MDR phenotype resistance. The MexAB-OprM is the only pump which is expressed at a level enough to allow intrinsic MDR in wild type *P. aeruginosa* strains. Mutations in *mex* R cause over-expression of MexAB-OprM efflux pump [36]. It is obvious in our study as shown in Figure-1; that all isolates exhibited the amplification of 637 bp which represent the *mex* R operon. The emergence of MDR *P. aeruginosa* is turning out a challenging issue in infection control schemes.

**Figure-2:**
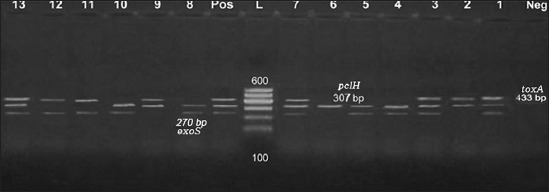
Multiplex polymerase chain reaction detection of virulence genes in *Pseudomonas aeruginosa* isolates showing: L: 100 bp DNA ladder. Lanes 1-13: *P. aeruginosa* isolates. Lane Pos.: Positive control; amplification of 270 bp represented exoS, 307 bp represented *plc*H, and 433 bp represented *tox*A. Lane Neg.: Negative control.

Figure-2 shows multiplex PCR detection of virulence genes in *P. aeruginosa* isolates *P. aeruginosa* is considered a potent opportunistic pathogen and this is contributed to its ability to produce arrays of virulence agents which aid the organism to attach, penetrate, disseminate, and inhibit phagocytosis, and chemotaxis [37]. The data obtained from Figure-2 revealed the multiplex PCR reaction of three genes amplification; *plc* H (307bp), *exo* S (270bp), and *tox* A (433 bp) which responsible for expression of cytotoxins, phospholipase, and enterotoxins as 12 (92.3%), 10 (76.9%), and 8 (61.5%), respectively. These results drew near the other previous investigations [38-42].

## Conclusion

*P. aeruginosa* could be implicated in sheep and goat infections, and the isolates showed high resistance to commonly used antibiotics as well as having numerous agents of virulence. A strict antibiotic policy and establishment of infection control programs will help to lower the incidence of resistance in *P. aeruginosa*.

## Authors’ Contributions

ASH and AND supervised the experiment. AMA and EAE shared in the collection of the samples, AND and HAA did the isolation and identification of microorganism. EAE performed the antibiotic susceptibility assay. HAA and AMA performed PCR. ASH and AND prepared and reviewed the manuscript. All authors read and approved the final manuscript.
